# In tumor cells, thyroid hormone analogues non-immunologically regulate PD-L1 and PD-1 accumulation that is anti-apoptotic

**DOI:** 10.18632/oncotarget.26143

**Published:** 2018-09-25

**Authors:** Hung-Yun Lin, Yu-Tang Chin, Ya-Jung Shih, Yi-Ru Chen, Matthew Leinung, Kelly A. Keating, Shaker A. Mousa, Paul J. Davis

**Affiliations:** ^1^ PhD Program for Cancer Molecular Biology and Drug Discovery, College of Medical Science and Technology, Taipei Medical University, Taipei, Taiwan; ^2^ Taipei Cancer Center, Taipei Medical University, Taipei, Taiwan; ^3^ Traditional Herbal Medicine Research Center of Taipei Medical University Hospital, Taipei, Taiwan; ^4^ TMU Research Center of Cancer Translational Medicine, Taipei Medical University, Taipei, Taiwan; ^5^ Department of Medicine, Albany Medical College, Albany, NY, USA; ^6^ Pharmaceutical Research Institute, Albany College of Pharmacy and Health Sciences, Rensselaer, NY, USA

**Keywords:** L-thyroxine, PD-1/PD-L1 immune checkpoint, cancer, tetrac

## Abstract

The PD-1/PD-L1 immune checkpoint involving tumor cells and host immune defense lymphocytes is a well-studied therapeutic target in oncology. That PD-1 and PD-L1 may have additional functions within tumor cells that are independent of the checkpoint is indicated by actions of a thyroid hormone analogue, L-thyroxine (T_4_), on these checkpoint components. Acting at a cell surface receptor on plasma membrane integrin αvβ3, T_4_ stimulates intracellular accumulation of PD-L1 in cancer cells. In these thyroid hormone-treated cells, T_4_-induced PD-L1 is non-immunologically anti-apoptotic, blocking activation of p53. Several laboratories have also described accumulation of PD-1 in a variety of cancer cells, not just immune defense lymphocytes and macrophages. Preliminary observations indicate that T_4_ stimulates intracellular accumulation of PD-1 in tumor cells, suggesting that, like PD-L1, PD-1 has non-immunologic roles in the setting of cancer. Where such roles are anti-apoptotic, thyroid hormone-directed cancer cell accumulation of PD-1 and PD-L1 may limit effectiveness of immunologic therapy directed at the immune checkpoint.

## INTRODUCTION

The programmed death-1 (PD-1)/PD-Ligand 1 (PD-L1) immune checkpoint has been extensively investigated [1-5]. PD-L1 production by tumor cells is a natural defense of cancer cells against host immune system destruction, downregulating the antitumor activity of immune T (killer) cells [1, 6-8]. Antibodies to PD-L1 and to PD-1 have been shown clinically to have important utility in the management of a variety of malignancies [9-15].

A non-antibody-based mechanism by which PD-L1 elaboration by tumor cells can be regulated involves thyroid hormone analogues [16, 17]. L-thyroxine (T_4_) is the principal product of the thyroid gland and is viewed as prohormone for the major intracellular thyroid hormone, 3,5,3’-triiodo-L-thyronine (T_3_). However, T_4_ has a panel of biological actions at a tumor cell surface receptor on plasma membrane integrin αvβ3 [18]. One of these actions downstream of the receptor is upregulation of transcription of PD-L1 [16]. A derivative of T_4_, tetraiodothyroacetic acid (tetrac), blocks this action of T_4_ initiated at αvβ3. We have proposed that tetrac, modified chemically to limit its actions to the exterior of tumor cells expressing αvβ3 [19], be tested as a non-antibody-based strategy to decrease or eliminate PD-L1 as a cancer cell defense [16].

## PD-L1 IN T_4_-TREATED TUMOR CELLS

Thyroid hormone as T_4_ in physiological free concentrations supports cancer cell proliferation and a number of survival pathways in tumor cells [20]. These cancer support actions are initiated by T_4_ at a receptor site on the extracellular domain of integrin αvβ3. While these actions are non-genomic at initiation—that is, they do not directly depend upon the nuclear receptors for thyroid hormone (TRs)—they may culminate downstream in transcription of specific genes [18], certain of which may involve TRs. These downstream effects are mediated by intracellular signal transduction systems, such as MAPK/ERK and PI3K. Unmodified or chemically converted to a nanoparticle, tetrac blocks actions of T_4_, including activation of MAPK and PI3K.

The involvement of ERK1/2 and PI3K in the enhancement of *PD-L1* gene expression [16, 21] caused us to search for possible involvement of T_4_ in the regulation of *PD-L1* transcription. Studied *in vitro* in human breast and colon cancer cell lines, *PD-L1* expression was enhanced by T_4_ [16] and complimented by accumulation of tumor cell PD-L1 protein by as much as 2.7-fold. Tetrac chemically bound to a poly lactic-co-glycolic acid (PLGA) nanoparticle, (Nanotetrac, nano-diamino-tetrac (NDAT)), substantially reduced the stimulatory effect of T_4_ on *PD-L1* gene expression and on abundance of cellular PD-L1 protein. In addition to its anti-apoptotic property, PD-L1 may also be a proliferative factor in certain cancer cells [22]. These results support the possibility that circulating host T_4_ is contributing to defensive activation in cancer cells of PD-L1.

The NDAT results indicated the feasibility of using a small molecule to modulate the PD-1/PD-L1 checkpoint by reducing the availability of PD-L1 [23]. This approach would also avoid the systemic adverse effects of PD-L1 antibody [24, 25] because actions of NDAT are limited to cancer cells and rapidly dividing endothelial cells that generously express αvβ3.

## NEWLY RECOGNIZED ROLES OF INTRACELLULAR PD-L1 AND PD-1 IN T_4_-TREATED CANCER CELLS

Against the background described, it is reasonable to ask whether PD-L1 has clinically undesirable intracellular effects that also may be avoided by downregulating the transcription of *PD-L1* with NDAT or similar compounds. Resveratrol, a stilbene with anticancer properties, can induce p53-dependent apoptosis in cancer cells by a mechanism that involves *nuclear* uptake of cyclooxygenase-2 (COX-2) [17, 26]. This was a novel role for COX-2.

Studied in the resveratrol-p53-COX-2 model in tumor cells exposed to T_4_, intracellular PD-L1 was found to be complexed with COX-2 in cytoplasm and no nuclear uptake of p53 and COX-2 occurred [17]. Therefore, resveratrol-induced apoptosis was inhibited. Thus, in addition to its function extracellularly as a ligand of T cell PD-1 at the PD-1/PD-L1 immune checkpoint and thus a defense against immune system destruction of cancer cells, PD-1 has an intracellular role as an inhibitor of inducible COX-2/p53-dependent apoptosis. This function is also a cancer cell survival mechanism for PD-L1. The results raise a set of questions that have not yet been addressed. For example, does the interaction of PD-L1 with inducible COX-2 in T_4_-treated tumor cells alter the function of COX-2 and reduce intracellular content of prostaglandins? Does this interaction model other protein-protein interactions in cytoplasm that are relevant to cancer cell defenses? The proteins could, for example, be signal transducing molecules or hormone-binding proteins such as nuclear thyroid hormone receptors (TRs) or estrogen receptors in cytoplasm.

**Figure 1:**
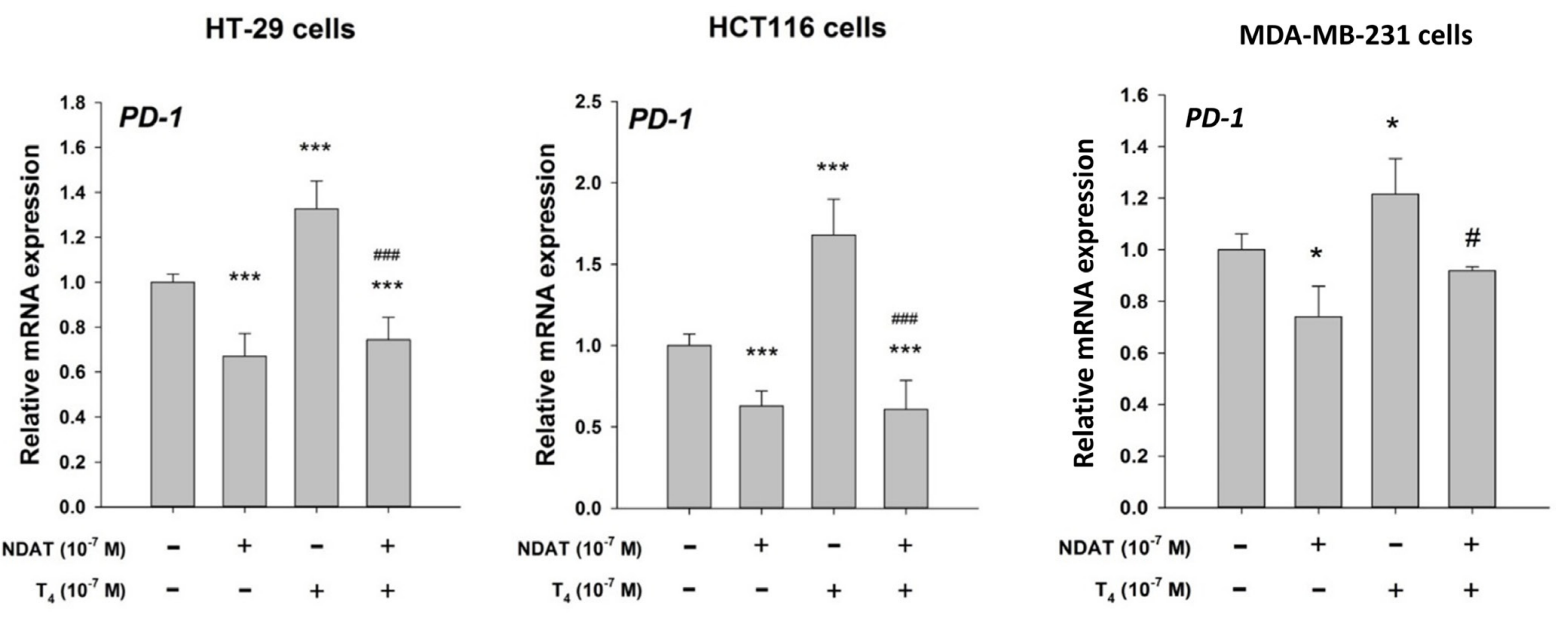
T_4_ induces *PD-1* mRMA expression in human colon cancer (HT-29, HCT116 and breast cancer (MDA-MB-231) cells *in vitro* Nano-diamino-tetrac (NDAT) inhibits actions of T_4_ that are initiated at plasma membrane integrin αvβ3 and has anticancer activity in the absence of T_4_. In this study, NDAT inhibited stimulatory activity of T_4_ on expression of *PD-1* mRMA and also reduced abundance of *PD-1* mRMA in the absence of T_4_. Materials, including cell lines, and methods used are as previously described [16]. Compared to control, *p <0.05, ***p <0.001; compared to T_4_, alone, #*p* <0.05, ###*p* 0.001.

The possibility that PD-1 has functions in cells other than T and B lymphocytes and macrophages has been suggested in reports from a number of laboratories that PD-1 is expressed by ovarian carcinoma cells [27], melanoma [28], small cell lung carcinoma [29], osteosarcoma cells [30] and murine lung carcinoma cells [31]. Physiological amounts of T_4_ induce expression of *PD-1* mRMA as well as accumulation of PD-1 protein in several human cancer cell lines (HY Lin: unpublished observations). NDAT blocks the action of T_4_ on the PD-1 axis in these cancer cells (Figure 1). Such preliminary studies provide no functional basis for the PD-1 response in human tumor cells, but it is known that injured, non-tumoral retinal ganglion cells (RGCs) express PD-1 [32], as do mouse RGCs scheduled to undergo apoptosis [33]. Thus, elaboration of PD-1 in cells other than lymphocytes and macrophages may be related to self-defense, e.g., apoptosis. This possibility requires systematic evaluation in other non-cancer cells.

## OVERVIEW

That a thyroid hormone analogue such as T_4_ can regulate intracellular concentrations of PD-1 and PD-L1 by a mechanism that is inhibitable by NDAT indicates that T_4_ is indeed biologically active and its activity is manifested via the hormone receptor on integrin αvβ3. T_4_ is the principal ligand of this receptor [20, 34], and NDAT at the concentration used is a specific inhibitor of thyroid hormone actions at αvβ3 [16]. The transcription of a large number of genes is regulated by this cell surface hormone receptor [35], and many of these are relevant to tumor cell proliferation, to tumor cell survival anti-apoptotic pathways [20] and to rapidly dividing endothelial cells and angiogenesis [36, 37]. We suggest that accumulation of intracellular PD-L1 and PD-1 in cancer cells offers another anti-apoptotic defense for tumor cells that is in a compartment inaccessible to clinically used antibodies to PD-1 and PD-L1 [23]. As noted above, accumulation of PD-L1 may occur in non-cancer cells that are at risk of apoptosis. Another role for PD-L1 involves regulation of angiogenesis [38]. Thus, distinct from their synergy in the function of the PD-1/PD-L1 immune checkpoint, these two moieties have functions as independent proteins. At least in part, these functions are regulated by thyroid hormone as T_4_.

Another issue is that resistance to apoptosis accompanies activation of the immune checkpoint in tumor cells [39]. Accumulation of PD-L1 and PD-1 within tumor cells exposed to T_4_ may be a component of the anti-apoptosis encountered in checkpoint activation.

T_4_ is known to have pro-angiogenic and anti-apoptotic properties [18], and the independent control by T_4_ via αvβ3 of PD-1 and PD-L1 production unrelated to the PD-1/PD-L1 immune checkpoint is consistent with roles already defined for T_4_. While further studies are required to determine how substantial the clinical contributions are of T_4_ to tumor-related angiogenesis and anti-apoptosis, elimination of T_4_ in patients with advanced cancers has shown stabilization of the disease and extended survival [40].
